# A Multi-Atlas-Based [18F]9-Fluoropropyl-(+)-Dihydrotetrabenazine Positron Emission Tomography Image Segmentation Method for Parkinson’s Disease Quantification

**DOI:** 10.3389/fnagi.2022.902169

**Published:** 2022-06-13

**Authors:** Yiwei Pan, Shuying Liu, Yao Zeng, Chenfei Ye, Hongwen Qiao, Tianbing Song, Haiyan Lv, Piu Chan, Jie Lu, Ting Ma

**Affiliations:** ^1^Department of Electronic and Information Engineering, Harbin Institute of Technology at Shenzhen, Shenzhen, China; ^2^Department of Neurology and Neurobiology, Xuanwu Hospital, Capital Medical University, Beijing, China; ^3^Chinese Institute for Brain Research (CIBR), Beijing, China; ^4^International Research Institute for Artificial Intelligence, Harbin Institute of Technology at Shenzhen, Shenzhen, China; ^5^Department of Radiology, Xuanwu Hospital, Capital Medical University, Beijing, China; ^6^Beijing Key Laboratory of Magnetic Resonance Imaging and Brain Informatics, Capital Medical University, Beijing, China; ^7^Mindsgo Life Science Shenzhen Co. Ltd., Shenzhen, China; ^8^National Clinical Research Center of Geriatric Disorders, Xuanwu Hospital Capital Medical University, Beijing, China; ^9^Advanced Innovation Center for Human Brain Protection, Capital Medical University, Beijing, China; ^10^Peng Cheng Laboratory, Shenzhen, China

**Keywords:** Parkinson’s disease, [18F]-FP-DTBZ, image segmentation, striatum subregion, SUVR quantification

## Abstract

**Objectives:**

[18F]9-fluoropropyl-(+)-dihydrotetrabenazine ([18F]-FP-DTBZ) positron emission tomography (PET) provides reliable information for the diagnosis of Parkinson’s disease (PD). In this study, we proposed a multi-atlas-based [18F]-FP-DTBZ PET image segmentation method for PD quantification assessment.

**Methods:**

A total of 99 subjects from Xuanwu Hospital of Capital Medical University were included in this study, and both brain PET and magnetic resonance (MR) scans were conducted. Data from 20 subjects were used to generate atlases, based on which a multi-atlas-based [18F]-FP-DTBZ PET segmentation method was developed especially for striatum and its subregions. The proposed method was compared with the template-based method through striatal subregion parcellation performance and the standard uptake value ratio (SUVR) quantification accuracy. Discriminant analysis between healthy controls (HCs) and PD patients was further performed.

**Results:**

Segmentation results of the multi-atlas-based method showed better consistency than the template-based method with the ground truth, yielding a dice coefficient of 0.81 over 0.73 on the full striatum. The SUVRs calculated by the multi-atlas-based method had an average interclass correlation coefficient (ICC) of 0.953 with the standardized result, whereas the template-based method only reached 0.815. The SUVRs of HCs were generally higher than that of patients with PD and showed significant differences in all of the striatal subregions (all *p* < 0.001). The median and posterior putamen performed best in discriminating patients with PD from HCs.

**Conclusion:**

The proposed multi-atlas-based [18F]-FP-DTBZ PET image segmentation method achieved better performance than the template-based method, indicating great potential in improving accuracy and efficiency for PD diagnosis in clinical routine.

## Introduction

Parkinson’s disease (PD) is one of the most common age-related neurodegenerative disorders (Braak et al., 2003; Rai and Singh, 2020; Rai et al., 2021a). An increasing number of evidence suggests that positron emission tomography (PET) imaging aiming at the assessment of the dopaminergic function supports a more accurate diagnosis of PD (Frey et al., 1996; Ravina et al., 2005; Pirker et al., 2010). Vesicular monoamine transporter type 2 (VMAT2) is the transporter responsible for the uptake and storage of monoamines. As VMAT2 imaging by PET provides reliable information for the degeneration of nigrostriatal dopaminergic neurons, a novel radiotracer named [18F]9-fluoropropyl-(+)-dihydrotetrabenazine ([18F]-FP-DTBZ) has been developed (Gilman et al., 1998; Okamura et al., 2010). Previous studies have revealed that the [18F]-FP-DTBZ uptake in the striatum is significantly associated with the severity of PD (Braak et al., 2003; Hsiao, 2014). Therefore, improvement in the PET image quantification method is important for objective assessment of PD progression and diagnosis especially in the early stage (Hsiao, 2014; Liu et al., 2018).

To quantify the PET images, the target-to-reference standard uptake value ratio (SUVR) is extensively used, by which the accuracy of identifying regions of interest (ROIs) directly affects the credibility of the quantification results (Clark et al., 2011; Fleisher et al., 2011; Wolk et al., 2012). Magnetic resonance image (MR)-based methods are widely applied to identify the target ROIs. With the assistance of structural MR images, segmentation results could achieve comparable accuracy with manual segmentation (Andersson et al., 1995; Kuhn et al., 2014). However, in clinical practice, the acquisition of extra MR images is expensive and inconvenient when patients take PET scans (Bailey et al., 2015). Template-based methods have been proposed to achieve ROI parcellation in the absence of MR data, by which target PET images are coregistered to a normalized PET template with predefined ROIs (Andersson et al., 1995; Chang et al., 2011; Dukart et al., 2011; Kuhn et al., 2014). However, in dopamine imaging, due to small ROI and heterogeneity of the tracer distribution, the single template strategy could not ensure the reliability of spatial normalization, which results in inevitable segmentation error and may introduce misestimate in the consequent quantitative analysis (van Rikxoort et al., 2010). In addition, most of the template-based methods have not reached quantification at the subregion level, though the distribution of dopamine receptors varies greatly among the striatal subregions, especially in the case of patients with PD (Staley and Mash, 1996; Martinez et al., 2003).

In this study, we proposed a multi-atlas-based [18F]-FP-DTBZ PET image segmentation method, which implements reliable MR-free ROI extraction for PET images. Comparisons between the multi-atlas-based, template-based method, and the gold standard were conducted on the segmentation and quantification level. Quantification results were further analyzed to reveal the most sensitive subregions in discriminating patients with PD. This would provide reliable information for PD diagnosis and facilitate efficient clinical workflow.

## Methods

### Subject Enrollment

A total of 2 datasets with 99 subjects recruited from the Xuanwu Hospital of Capital Medical University were included in this study. All subjects underwent a PET and a high-resolution MR scan of the brain. UI dataset with PET/MR (scanned by United Imaging uPMR790) includes 30 HCs and 38 PDs. GE dataset with PET/MR (scanned by GE Healthcare) includes 11 HCs and 20 PDs. The PDs were diagnosed according to the MDS clinical diagnostic criteria for PD (Postuma and Berg, 2017). The study was approved by the Institutional Review Board of Xuanwu Hospital. Written informed consent was obtained from all participants prior to the study procedure.

### Data Acquisition

#### UI Dataset

Magnetic resonance imaging data acquisition was performed using a hybrid 3.0-T PET/MR scanner (uPMR790, UIH, Shanghai, China) with a 24-channel head/neck coil. A 3D T1-weighted imaging (T1WI) data were collected from all participants with the following parameters: TR/TE: 7.86/3.2 ms; flip angle: 10; FOV: 230 mm × 256 mm; voxel size: 0.5 mm × 0.5 mm × 0.67 mm. Scanning parameters of [18F]-FP-DTBZ PET imaging were as follows: field of view = 300 mm; voxel size = 1.17 mm × 1.17 mm × 1.4 mm. All patients with PD were scanned during their off-state condition (12 h after the last medication).

#### GE Dataset

Positron emission tomography/MRI examinations were performed on an integrated simultaneous Signa PET/MR scanner (GE Healthcare). The scan began 90 min following an intravenous bolus injection of around 222 MBq (6 mCi) of [18F]-FP-DTBZ and lasted for 15 min. The PET bed position included a simultaneous 18-s 2-point Dixon scan for MRI-based attenuation correction as well as additional diagnostic 3D T1 BRAVO MR images with scanning parameters as follows: TR = 7.9 ms; TE = 3.6 ms; acquisition matrix = 232 × 224; acquired spatial voxel resolution 1.00 mm × 1.00 mm × 1.00 mm with a data acquisition time of 4 min and 17 s. Scanning parameters of PET imaging were as follows: field of view = 25 cm; matrix size = 192 × 192; voxel size = 1.82 mm × 1.82 mm × 2.78 mm; 89 slices. Attenuation correction, scatter correction, random correction, and dead-time correction were performed as well.

### Positron Emission Tomography Segmentation Pipeline

**FIGURE 1:**
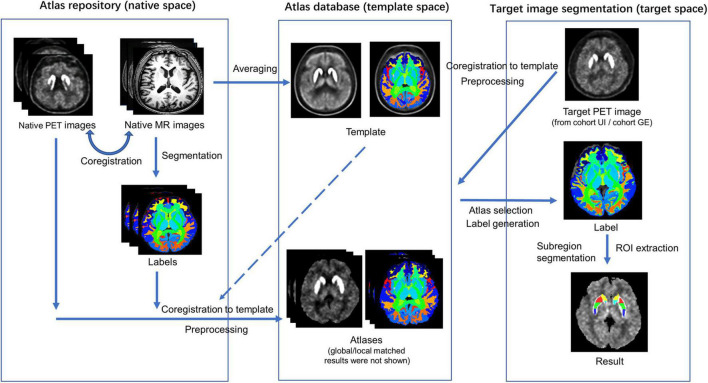
Schematic representation of the procedure for the automatic multi-atlas-based PET image segmentation method.

The multi-atlas-based [18F]-FP-DTBZ PET image segmentation and analysis method were developed by the procedure illustrated in Figure 1. A total of 20 subjects including 10 HCs and 10 patients with PD were selected randomly from the UI dataset as the atlas repository (9 men and 11 women). The remaining subjects in the UI dataset were used for testing, named cohort UI. GE dataset was used for reproducibility experiment, named cohort GE.

#### Atlas Creation

First, the rigid coregistration was performed on the MR images to the corresponding PET images for each individual in the atlas repository. A PET template was constructed by averaging all PET images using the multivariate template construction method by Advanced Normalization Tools (ANTs^1^) (Avants et al., 2009). An MR template was generated using the same method and coregistered to the PET template. Individual MR images, as well as the MR template, were segmented using Brain Label,^2^ which executes brain parcellation of T1 image based on pre-selection strategy and multiple atlas likelihood fusion algorithm (Tang et al., 2013; Wu et al., 2016). By applying the Brain Label, whole-brain label images consisting of 283 regions, including the caudate, putamen, nucleus accumbens, and cerebellum, were generated.

After that, the PET images were coregistered to the PET template using the non-linear registration method by ANTs. The transform matrix and normalization parameters generated by these two steps were also applied to the label images. A manual check on segmentation accuracy for each participant was performed by a senior neurologist with 7-year experience in nuclear image processing.

Several image preprocessing methods were applied to the PET images in the template space, including skull stripping, partial volume effect correction, smoothing, and histogram specification. Each individual skull stripped PET image was corrected for partial volume effect by PETPVC, with kernel size = 6.0 mm × 6.0 mm × 6.0 mm, number of iterations = 10, number of deconvolution iterations = 10, alpha value = 1.5, stopping criterion = 0.01 (Thomas et al., 2016; Alavi et al., 2018). The VOI mask required by PETPVC was extracted from the label of the template. An isotropic Gaussian kernel of 3.7 mm × 3.7 mm × 6.6 mm full width at half maximum was applied for smoothing (Tournier et al., 2019). Global histogram specification was applied to transform the histogram of the individual PET images to the histogram of the template, followed by local histogram specification on the ROI of the striatum. Through histogram specification, PET image intensity was normalized to facilitate further comparison. Supplementary Figure 1 shows the examples of histogram specifications.

Finally, an atlas database including a PET template and 20 atlases was obtained to provide segmentation reference for target PET images. Each atlas contained a PET image in the template space with a corresponding label image and its global as well as local histogram-specified results.

#### Multi-Atlas-Based Positron Emission Tomography Segmentation

Target PET images in cohort UI were coregistered to the template space and followed a similar preprocessing procedure as PET atlases (skull stripping, smoothing, and global and local histogram specification) for further evaluation. Mean square error (MSE) was applied to measure the similarity between target PET images and atlases. For each target, five best-matched atlases from the atlas database were selected by following two steps. In the first step, MSE between each atlas’ global-specified PET image and the target’s global-specified PET image was calculated. A total of 10 atlases with the least MSE values were selected as the candidates for the second step. In the second step, five best-matched atlases were selected based on MSE calculated between local-specified regions (i.e., the striatum) on PET images.

The segmentation results were produced by merging the label images of the best-matched atlases. The joint label fusion (JLF, operated by ANTs) was performed to fuse the obtained multiple labels of the PET image for each target participant (Avants et al., 2009). First, the chosen PET images were coregistered to the target PET image. Using the warping parameters, label images of the chosen atlases were warped to the target PET space and subsequently fused into one label image. Comparisons were conducted between the multi-atlas-based method and the conventional template-based method. The template-based method shared the same PET template as well as its label generated in section “Atlas Creation,” VOI was selected from the whole-brain label image, and the target PET image was coregistered to the template directly for VOI extraction.

#### Subregion Segmentation

**FIGURE 2:**
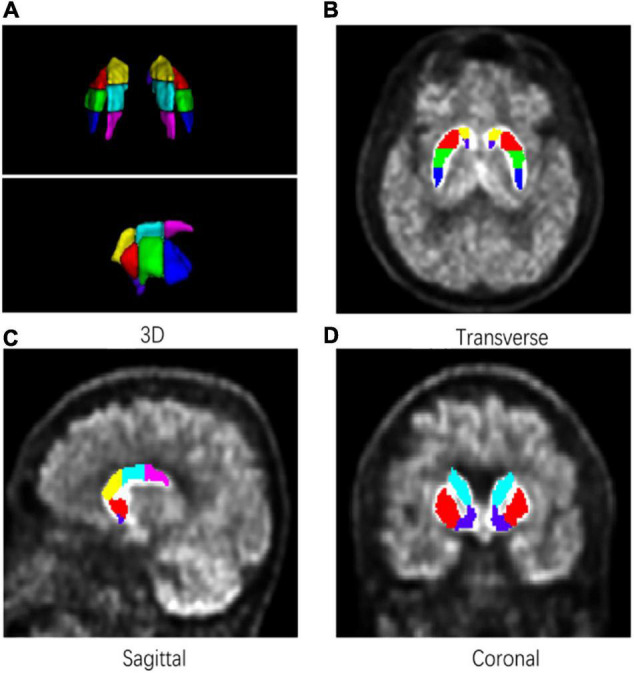
An example of a subregion segmentation result. Bilateral putamen and caudate were divided into three equal parts in length. **(A)** 3D. **(B)** Transverse. **(C)** Sagittal. **(D)** Coronal.

A detailed subregion segmentation criterion based on the topographic structural information was adopted to find prominent parts of the striatum for discriminating patients with PD from HCs. The putamen and the caudate were divided into anterior, median, and posterior parts, respectively. Using the median point of the whole putamen pixels under the coronal axis, a sagittal plane was established to separate the striatum into left and right parts. Then, the bilateral putamen and caudate were further segmented into three equal parts along the line determined by their front-end and back-end points under the sagittal axis. Finally, the striatum was segmented into seven subregions (bilaterally, including the anterior putamen, median putamen, posterior putamen, anterior caudate, median caudate, posterior caudate, and nucleus accumbens). Figure 2 shows a sample of subregion segmentation result in 2D and 3D images to illustrate our subregion segmentation criterion. The total time required to segment a target PET image was about 20 min [CPU: Intel X(R) CPU E5-2620 v4 @ 2.10 GHz].

### Standard Uptake Value Ratio Evaluation

Standardized uptake value ratios of all seven subregions of the striatum on each PET image were calculated by dividing the mean counts per voxel in the target subregions by the mean counts per voxel in the reference region (Cerebellum):


(1) $$\mathrm{SUVR}=\frac{\mathrm{target}\mathrm{uptake}}{\mathrm{reference}\mathrm{uptake}}$$

The result of the MR-based method was applied as the ground truth. MR images for cohort UI subjects were segmented by Brain Label and coregistered to PET images. The subregion segmentation criterion was the same as mentioned before, and the final results were manually checked by the experienced neurologist.

### Statistical Evaluation

Dice coefficient was applied to measure the similarity of segmentation results between the multi-atlas-based method and the MR-based method (Dice, 1945). It is computed by:


(2) $$\mathrm{dice}=\frac{2\left|A\cap B\right|}{\left|A\right|+\left|B\right|}$$

where A and B are two different segmentation results, respectively, and |⋅| represents the number of voxels within the segmentation result. A larger dice coefficient means better overlap. For larger structures, values above 0.8 are usually accepted as successful results while for smaller structures, values greater than 0.7 are preferred (Xiao et al., 2015).

The two-way mixed effect interclass correlation coefficients (ICCs) were calculated to identify the consistency between the SUVRs obtained by the two segmentation methods (Shrout and Fleiss, 1979). ICC is calculated by:


(3) $$\mathrm{ICC}=\frac{\sigma_{s}^{2}}{\sigma_{s}^{2}+\sigma_{s}^{2}}$$

where σ_*s*_ denotes variance caused by differences between the segmentation methods and σ_*s*_ denotes variance caused by differences between the values in the segmentation results.

Statistical analysis of demographic details between groups was performed using a two-sample *t*-test and effect size. The *t*-test was further applied to compare SUVRs between HCs and patients with PD. Additionally, *p* < 0.05 was determined significant in our statistical results.

## Results

### Demographic Results

Table 1 summarizes the demographic details of participants from UI scanner. Descriptive data are presented as the mean ± standard deviation (SD) for continuous variables and percentage for dichotomous variables. No significant group differences were found in sex and age. In the PD group, mean UPDRS-III score was 21.5 (SD = 11.8) and the median of Hoehn and Yahr stage was 2 (range 1–5). The demographic details of participants from GE scanner are shown in Supplementary Table 1.

**TABLE 1:** Demographic details of participants from UI scanner.

Group	Sample size	Sex (M/F)	Age (years)	UPDRS-III	HY
HC_UI	30	13/17	56.8 ± 10.5	–	–
PD_UI	38	18/20	55.8 ± 15.6	21.5 ± 11.8	1.9 ± 0.9

*HC_UI, healthy control from UI scanner; PD_UI, Parkinson’s disease from UI scanner.*

### Segmentation Performance Evaluation

We compared the segmentation accuracy of the striatum subregions between the multi-atlas-based and template-based methods on cohort UI. As shown in Table 2, the multi-atlas-based method showed better segmentation performance. The mean dice coefficients of the whole striatum, caudate, and putamen all showed excellent accuracy (dice >0.8). The nucleus accumbens had the worst performance due to its smaller size. Compared to the multi-atlas-based method, the template-based method had a general performance degradation of 8%, which indicated the multi-atlas strategy held better performance than the single template strategy.

**TABLE 2:** Dice coefficients of two label generation methods in ROI regions.

Label generation methods	ROI regions			
	Nucleus accumbens	Caudate	Putamen	Striatum
Template-based	0.60 ± 0.04	0.73 ± 0.06	0.75 ± 0.04	0.73 ± 0.04
Multi-atlas-based	0.71 ± 0.05	0.81 ± 0.04	0.83 ± 0.05	0.81 ± 0.04

*Data are presented as mean value ± SD.*

**FIGURE 3:**
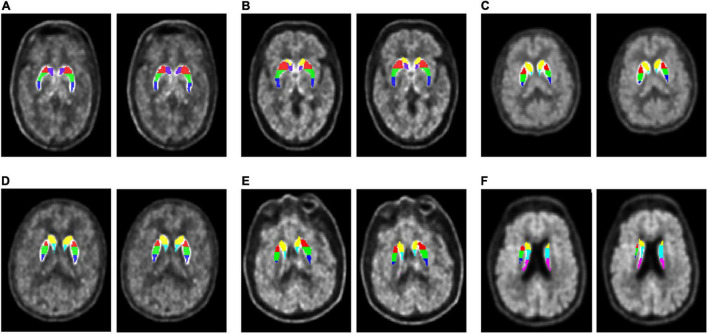
Segmentation results comparison. Each subgraph consists of a subject’s original PET image, superimposed with seven subregion labels obtained by two segmentation methods, the multi-atlas-based method (left) and the MR-based method (right). Both PDs and HCs from cohort UI and GE were selected to demonstrate their segmentation effects at different cross-sectional slices. **(A)** A 46-year-old HC (male) in cohort UI case 4. **(B)** A 56-year-old PD (female) in cohort UI case 20. **(C)** A 48-year-old PD (female) in cohort GE case 3. **(D)** A 59-year-old HC (female) in cohort UI case 12. **(E)** A 61-year-old PD (male) in cohort UI case 32. **(F)** A 75-year-old PD (male) in cohort GE case 31.

Figure 3 presents the subregions obtained by the multi-atlas-based method and the MR-based method. For each case, the result of the multi-atlas-based method was shown on the left whereas the result of the MR-based was shown on the right. The backgrounds were the native PET images. Segmentation results of the multi-atlas-based method showed good agreement with the MR-based method in varied conditions and slices.

### Quantification Performance Evaluation

**FIGURE 4:**
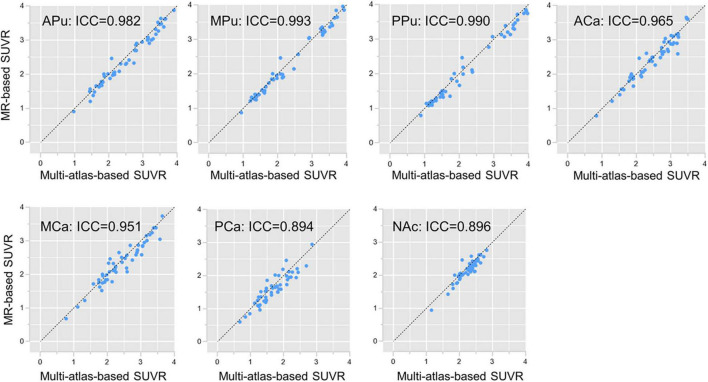
Subregion SUVR correlations. Subregion SUVR correlations between the multi-atlas-based PET segmentation and the MR-based segmentation in cohort UI. The horizontal axis is the SUVR of each subregion separated by the multi-atlas-based PET segmentation, whereas the vertical axis is the SUVR of each subregion separated by the MR-based segmentation.

The subregion SUVR values were calculated according to the segmentation results generated by the multi-atlas-based method and the MR-based method, respectively. The correlation of results obtained by these two analyses was calculated and visualized in Figure 4. As demonstrated, SUVR of all subregions showed great consistency with an average ICC of 0.953. In contrast, the average ICC between the template-based method and the MR-based method was 0.815 (Supplementary Figure 2).

### Standard Uptake Value Ratio Level in Diagnostic Groups

**FIGURE 5:**
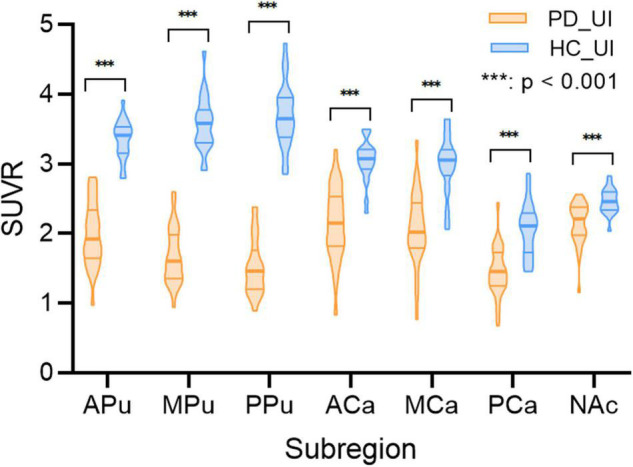
Subregion [18F]-FP-DTBZ SUVRs in cohort UI. In the violin plot, the central mark indicates the median, and the bottom and top edges indicate the 25th and 75th percentiles. HC_UI, healthy control from UI scanner; PD_UI, Parkinson’s disease from UI scanner. Significance: ***p < 0.001 *t*-test analysis.

Figure 5 and Table 3 show the SUVRs of [18F]-FP-DTBZ in the subregions of PD and HC groups from cohort UI. The SUVRs of HCs were generally higher than that of patients with PD and showed significant differences in all of the subregions (all *p* < 0.001). The SUVRs of the median putamen and posterior putamen in patients with PD could be separated clearly from HCs without any overlap between the two groups. The *t*-test demonstrated SUVR values to be significantly different in all of the subregions for HCs and patients with PD (*p* < 0.001). Supplementary Table 2 shows the effect size of the multi-atlas-based method and the template-based method. In most subregions, the multi-atlas-based method obtained better divergence in discriminating HC and patients with PD.

**TABLE 3:** [18F]9-fluoropropyl-(+)-dihydrotetrabenazine SUVRs in seven subregions in PDs and HCs from cohort UI.

	Subregion	APu	MPu	PPu	ACa	MCa	PCa	NAc
Statistical description SUVR mean (SD)	HC_UI	3.34 (0.29)	3.57 (0.41)	3.67 (0.47)	3.04 (0.31)	2.97 (0.43)	2.03 (0.39)	2.46 (0.18)
	PD_UI	1.99 (0.46)	1.67 (0.40)	1.52 (0.40)	2.14 (0.53)	2.07 (0.53)	1.45 (0.36)	2.14 (0.31)
*t*-Test analysis	*t*	11.52	15.94	17.09	6.75	6.21	5.36	4.18
	*p*	<0.001 ***	<0.001 ***	<0.001 ***	<0.001 ***	<0.001 ***	<0.001 ***	<0.001 ***

HC_UI, healthy control from UI scanner; PD_UI, Parkinson’s disease from UI scanner. Significance: ***p < 0.001.

### Reproducibility Experiments

Reproducibility experiments were performed on cohort GE. The multi-atlas-based PET image segmentation method and the MR-based method were applied to extract the ROI subregions. The dice coefficient of the two methods reached 0.792 on the whole striatum and 0.816, 0.788, and 0.652 on the putamen, caudate, and nucleus accumbens, respectively. SUVR of all subregions calculated by the multi-atlas-based method and the MR-based method is compared in Supplementary Figure 3 having an average ICC of 0.969. Supplementary Figure 4 and Supplementary Table 3 demonstrated the SUVR comparison between HCs and PDs in cohort GE and showed a similar pattern as cohort UI, indicating that the multi-atlas-based method could be applied for multi-center use.

## Discussion

In this study, we proposed a multi-atlas-based PET segmentation method that achieved reliable segmentation performance in an MR-free situation. Analysis on [18F]-FP-DTBZ PET quantification showed great potential for our method in PD diagnosis. The current PET image analysis methods mainly include the manual method, MR-based method, and MR-free template-based method. Our multi-atlas-based method was fully automatic and did not rely on MR images. Thus, it was operator-independent and had great reproducibility with high processing efficiency. As compared to the template-based method, our proposed method used an atlas database rather than a single template, which provided a higher parcellation accuracy. In the previous studies, the PET template was usually generated from HCs or adapted from existing results such as Montreal Neurological Institute (MNI) standard space, and target PET images were coregistered to the PET template directly (Evans et al., 1993). Given that the binding of [18F]-FP-DTBZ was affected by VMAT2 distribution, PET images of different disease severity would present different intensity distributions according to VMAT2 density (Lin et al., 2011). However, the coregistration method could not work well when the image intensity did not reflect the real structure information. So, coregistration error would be introduced if only one template is referenced. The multi-atlas selection strategy could automatically select the most matched atlases using both global and local information, thus avoiding potential segmentation degeneration caused by coregistration with inappropriate images. By comparison, the multi-atlas-based method showed better accuracy than the template-based method (Table 2).

Our method further segmented the striatum into seven subregions. The putamen and the caudate were divided into three parts, respectively. Our study showed that the reduction of VMAT2 integrity among patients with PD varies in different parts of the striatum. The improvement in the segmentation method helped to further distinguish patients with PD from HCs. Analysis of SUVR showed that all subregions of patients with PD exhibited different levels of lower VMAT2 densities than HCs, suggesting presynaptic nigrostriatal dysfunction in PD (Lung et al., 2018). The most affected subregion was the posterior putamen, followed by the rest parts of the putamen and subregions in the caudate, which was in line with the previous study (Okamura et al., 2010; Hsiao, 2014). SUVRs in the median putamen and posterior putamen had the best performance to discriminate patients with PD from HCs.

The multi-atlas-based method also showed potential in processing datasets from other institutions and was expected to be applicable to other PET modalities (Bui et al., 2020). Reproducibility experiments indicated that our method could achieve guaranteed segmentation and quantification results on a different scanner. In this study, [18F]-FP-DTBZ PET was selected as our imaging modality because VMAT2 has a high density in striatal regions and shows low sensitivity to drug effects (Lee et al., 2000; Sun et al., 2012). But the multi-atlas-based method could be applied to other PETs targeting DAT or AADC as well (de Natale et al., 2018). Since the generation of PET images followed the same principle, our method could also be utilized for some other neurodegenerative diseases which could be examined by PET such as AD.

Several limitations in this study should be mentioned. First, a large number of subjects, as well as samples from other imaging centers for further validation, are warranted. Due to the limited sample size, our current atlas database only included 20 cases. Although a significant segmentation performance improvement has been achieved, more atlases were expected to provide better segmentation accuracy since more PET distribution patterns might be included. Second, our subregion segmentation criterion was restricted to topographic structural information. Through significant difference has been found in some of the subregions, the combination of structural connectivity and functional information might provide more insights into dopamine function and contribute to better subdivision (Tziortzi et al., 2014; Rai et al., 2020, 2021b). Finally, future work to establish the correlation between the analysis results and the clinical disease severity is needed. As we did not include H–Y stage information of subjects in the study, further studies focusing on evaluating the performances of our proposed method in different severities of PD are warranted.

## Conclusion

In this study, a multi-atlas-based [18F]-FP-DTBZ PET image segmentation method was proposed for PD quantification and showed better performance than the template-based method. The application analysis in patients with PD suggests that the proposed method has potential value for improving the accuracy and efficiency of PD diagnostic in clinical routine.

## Data Availability Statement

The datasets presented in this article are not readily available because it is for institutional use only. Requests to access the datasets should be directed to TM, tma@hit.edu.cn.

## Ethics Statement

The studies involving human participants were reviewed and approved by the Institutional Review Board of Xuanwu Hospital. The patients/participants provided their written informed consent to participate in this study. Written informed consent was obtained from the individual(s) for the publication of any potentially identifiable images or data included in this article.

## Author Contributions

TM conceived and supervised the project. HQ and TS helped the data acquisition. YP, SL, YZ, and CY analyzed the data. YP drafted the manuscript. YZ created the figures. CY and HL revised the manuscript. SL, PC, and JL provided the insight on clinical aspects. TM, JL, and PC gave final approval to the manuscript. All authors read and approved the final manuscript.

## Conflict of Interest

HL was employed by Mindsgo Life Science Shenzhen Co. Ltd. The remaining authors declare that the research was conducted in the absence of any commercial or financial relationships that could be construed as a potential conflict of interest.

## Publisher’s Note

All claims expressed in this article are solely those of the authors and do not necessarily represent those of their affiliated organizations, or those of the publisher, the editors and the reviewers. Any product that may be evaluated in this article, or claim that may be made by its manufacturer, is not guaranteed or endorsed by the publisher.

## Footnotes

1
http://picsl.upenn.edu/software/ants

2
http://brainlabel.org

## Abbreviations

ANTs: Advanced Normalization Tools
[18F]-FP-DTBZ: [18F]9-fluoropropyl-(+)-dihydrotetrabenazine
HC: healthy control
ICC: interclass correlation coefficient
JLF: joint label fusion
MSE: mean square error
PD: Parkinson’s disease
SUVR: standard uptake value ratio
VMAT2: vesicular monoamine transporter type 2.
