# Supplementary material for: A Multi-Atlas-Based [18F]9-Fluoropropyl-(+)-Dihydrotetrabenazine Positron Emission Tomography Image Segmentation Method for Parkinson’s Disease Quantification
Source: Front Aging Neurosci. 2022 Jun 13;14:902169. doi: 10.3389/fnagi.2022.902169 (PMC9234266; doi:10.3389/fnagi.2022.902169)
Supplement: Supplementary file 1 [file Data_Sheet_1.docx]

Supplementary Material

# Supplementary Figures


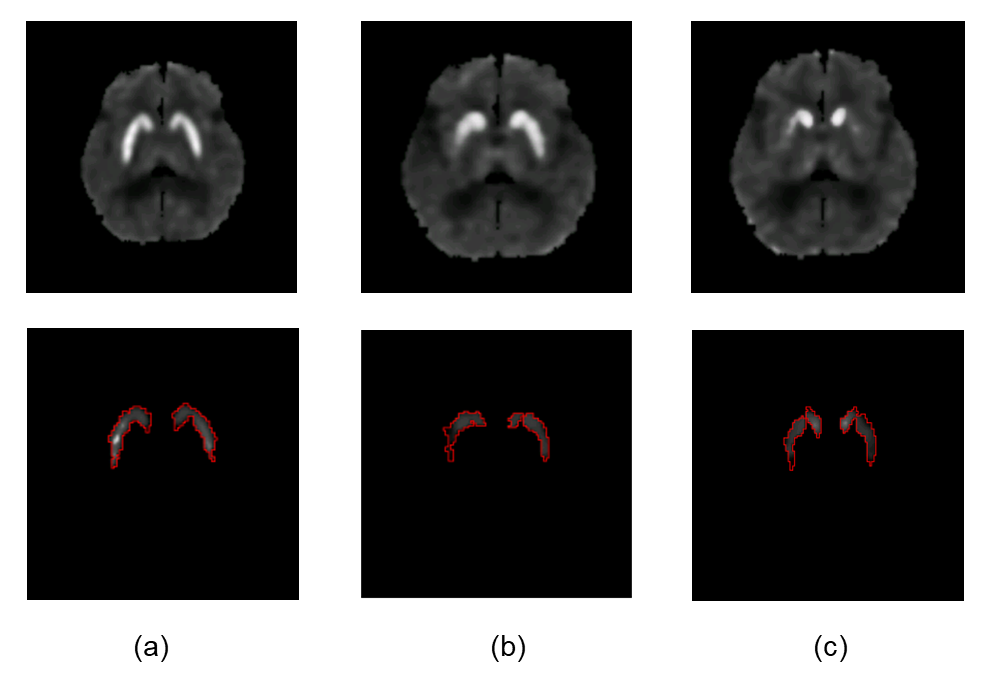


**Supplementary Figure 1.** Examples of global (up) and local (down) histogram specification results in the atlas database. In local histogram specification results, ROI was specifically indicated. (a) A 58-year-old HC (male) in atlas database case1. (b) An 85-year-old PD (male) in atlas database case11. (c) A 60-year-old PD (male) in atlas database case15.


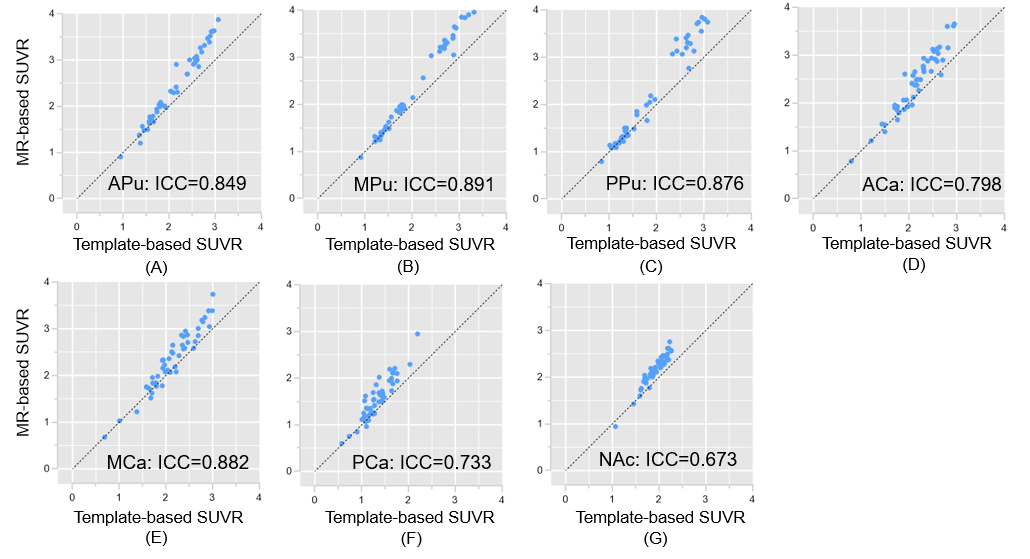


**Supplementary Figure 2.** Subregion SUVR correlations between the template-based PET segmentation and the MR-based segmentation in cohort UI. The horizontal axis is the SUVR of each subregion separated by the template-based PET segmentation, while the vertical axis is the SUVR of each subregion separated by the MR-based segmentation.


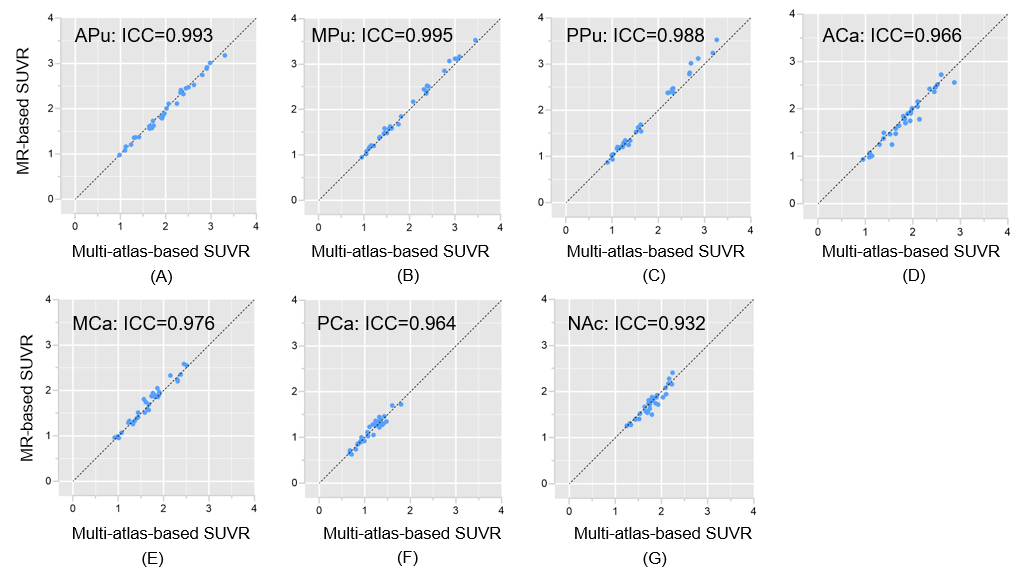


**Supplementary Figure 3.** Subregion SUVR correlations. Subregion SUVR correlations between the multi-atlas-based PET segmentation and the MR-based segmentation in cohort GE. The horizontal axis is the SUVR of each subregion separated by the multi-atlas-based PET segmentation, while the vertical axis is the SUVR of each subregion separated by the MR-based segmentation.


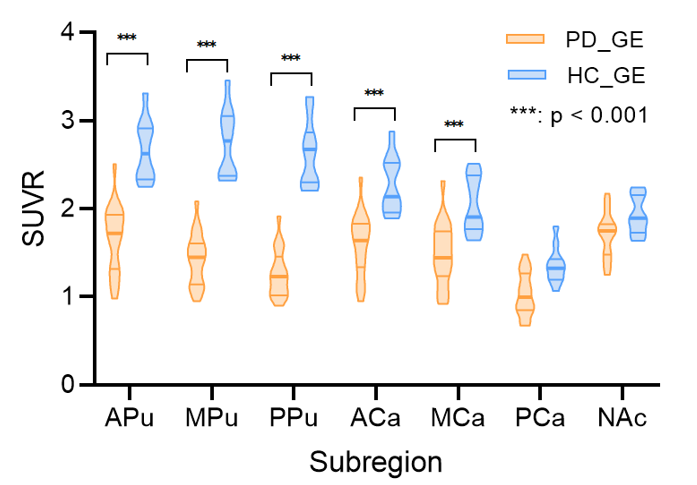


**Supplementary Figure 4.** Subregion [18F]-FP-DTBZ SUVRs in cohort GE. In the violin plot, the central mark indicates the median, and the bottom and top edges indicate the 25th and 75th percentiles. HC_GE = Healthy control from GE scanner; PD_GE = Parkinson’s disease from GE scanner. Significance: *** p < 0.001 t-test analysis.

# Supplementary Tables

**Supplementary Table 1.** Demographic details of participants from GE scanner.

| Group | Sample size | Sex(M/F) | Age(years) |
| --- | --- | --- | --- |
| HC_GE | 11 | 8/3 | 55.3 ± 14.4 |
| PD_GE | 20 | 10/10 | 51.5 ± 10.9 |

HC_GE = Healthy control from GE scanner; PD_ GE = Parkinson’s disease from GE scanner.

**Supplementary Table 2.** Effect size of SUVRs between HC and PD patients from cohort UI.

| Methods | Effect size | ROI regions | | | | | | |
| --- | --- | --- | --- | --- | --- | --- | --- | --- |
|  |  | APu | MPu | PPu | ACa | MCa | PCa | NAc |
| Multi-atlas-based | Cohen’s d | 3.64 | 4.89 | 5.19 | 2.14 | 1.95 | 1.68 | 1.30 |
|  | R | 0.88 | 0.93 | 0.93 | 0.73 | 0.70 | 0.64 | 0.54 |
| Template-based | Cohen’s d | 3.59 | 5.01 | 5.00 | 1.93 | 1.87 | 1.62 | 1.10 |
|  | R | 0.87 | 0.93 | 0.93 | 0.69 | 0.68 | 0.63 | 0.48 |

**Supplementary Table 3.** [18F]-FP-DTBZ SUVRs in 7 subregions in PDs and HCs from cohort GE.

|  | Subregion | APu | MPu | PPu | ACa | MCa | PCa | NAc |
| --- | --- | --- | --- | --- | --- | --- | --- | --- |
| Statistical description  SUVR Mean (SD) | HC_GE | 2.62  (0.34) | 2.83  (0.40) | 2.78  (0.40) | 2.20  (0.34) | 2.08  (0.35) | 1.35  (0.22) | 1.92  (0.24) |
|  | PD_GE | 1.62  (0.37) | 1.43  (0.30) | 1.24  (0.22) | 1.54  (0.39) | 1.51  (0.37) | 1.02  (0.26) | 1.63  (0.27) |
| t-test  analysis | T | 7.45 | 11.03 | 13.76 | 4.66 | 4.20 | 3.56 | 2.98 |
|  | p | <0.001  *** | <0.001  *** | <0.001  *** | <0.001  *** | <0.001  *** | 0.001  ** | 0.006  ** |

HC_GE = Healthy Control from GE scanner; PD_GE = Parkinson’s Disease from GE scanner. Significance: ^***^ p < 0.001; ^**^ p < 0.01; ^*^ p < 0.05
